# Differential expression of M3 muscarinic receptors in progressive colon neoplasia and metastasis

**DOI:** 10.18632/oncotarget.15500

**Published:** 2017-02-18

**Authors:** Kunrong Cheng, Aaron C. Shang, Cinthia B. Drachenberg, Min Zhan, Jean-Pierre Raufman

**Affiliations:** ^1^ Veterans Affairs Maryland Healthcare System, Baltimore VA Medical Center, Baltimore, MD, USA; ^2^ Department of Medicine, Division of Gastroenterology and Hepatology, University of Maryland School of Medicine, Baltimore, MD, USA; ^3^ Department of Pathology, University of Maryland School of Medicine, Baltimore, MD, USA; ^4^ Department of Epidemiology and Public Health, University of Maryland School of Medicine, Baltimore, MD, USA; ^5^ Marlene and Stewart Greenebaum Comprehensive Cancer Center, University of Maryland School of Medicine, Baltimore, MD, USA

**Keywords:** colon neoplasia, metastases, muscarinic receptors, matrix metalloproteinase-1, receptor expression

## Abstract

M3 muscarinic receptor (M3R) activation promotes colon cancer cell proliferation, migration, and invasion *in vitro*. Although over-expression of *CHRM3*, the gene encoding M3R, is reported in primary colon cancers, expression of M3R itself has not been studied in colon neoplasia. We compared M3R expression in normal colon to colon adenomas, and primary and metastatic colon cancers. Compared to adjacent normal colon, *CHRM3* expression was increased up to 128-fold in 10 of 18 consecutive surgical cancer specimens (56%) and associated with metastatic spread (*P* < 0.05). To analyze M3R protein expression we interrogated 29 consecutive paraffin-embedded colon adenocarcinomas and adjacent normal colon using a specific anti-M3R antibody and immunoperoxidase staining. This revealed weak M3R expression in normal colonocytes, primarily on basolateral surfaces. In contrast, in 25 of 29 cancer tissues (86%) we observed both cytoplasmic and plasma membrane over-expression of M3R; compared to normal epithelium, mean M3R staining intensity was increased more than two-fold in colon cancer (*P* < 0.001). M3R staining was also increased in 22 colon adenomas compared to adjacent normal colon (*P* < 0.001). In contrast, M3R staining intensity was not increased in lymph node or liver metastases. These findings suggest M3R expression plays an important role in early progression and invasion of colon neoplasia but is less important once tumors have spread.

## INTRODUCTION

Of five known muscarinic receptor subtypes, three that stimulate cellular signaling by means of phospholipid turnover (M1R, M3R, and M5R) are conditional oncogenes when expressed in cells capable of proliferation [1]. Of these three receptor subtypes, M3R are expressed widely in the gut and *CHRM3*, the gene encoding M3R, is reported to be uniquely over-expressed in colon cancer [2, 3].

Muscarinic receptors are activated when bound by acetylcholine released from neurons or non-neuronal cells, including colon cancer cells [4]. M3R can also be activated by bile acids [5, 6]; fecal bile acids have been implicated as tumor promoters by both epidemiological evidence in humans and bile acid-feeding studies in rodents [7, 8]. In mice lacking a key intestinal bile acid transporter, we found increased spillage of bile acids into the colon was associated with augmented colon neoplasia and increased expression of M3R, a surrogate for M3R activation [9, 10].

*In vitro* studies using human colon cancer cells reveal that muscarinic receptor agonists stimulate cell proliferation, survival, migration, and invasion by complex mechanisms involving interacting post-M3R signaling pathways as well as cross-talk which activates epidermal growth factor receptors (EGFR) and a different set of post-receptor signaling cascades [11]. In particular, rapid, reversible activation of ERK1/2 regulates colon cancer cell proliferation and PI3K/AKT activation regulates cell survival and resistance to radiation [11, 12]. In animal models relevant to sporadic and genetic human colon cancer, M3R activation stimulates colon cancer growth [13] and M3R deficiency attenuates tumor formation [14, 15]. Collectively, these findings support an important role for M3R expression and activation in the progression of colon neoplasia.

Despite these intriguing observations, the expression of M3R protein in the normal human large intestine has not been compared to that in colon adenocarcinomas or other stages of colon neoplasia. To address this gap in knowledge we compared M3R expression in normal colon epithelium to that in colon adenomas, and primary and metastatic colon cancers. To avoid inter-individual variation, whenever possible we used matched specimens of normal colon along with primary and metastatic lesions from the same patient. Also, since *in vitro* studies show that M3R activation strongly induces expression of matrix metalloproteinase-1 (*MMP1*), an enzyme that degrades extracellular matrix [16] and whose expression correlates with advanced colon cancer stage, metastasis and poor prognosis [17, 18], we explored the association of *CHRM3*/M3R and MMP1 expression in primary colon cancers.

As reported herein, we observed that measuring *CHRM3* mRNA expression alone underestimates the extent to which M3R is over-expressed in colon neoplasia. Moreover, whereas M3R expression appears to be important for the progression of primary adenomas and adenocarcinomas and the development of metastatic disease, it appears less important in established lymph node and liver metastases. Finally, although MMP1 expression was robustly increased in almost all primary colon cancers, we were unable to demonstrate a quantitative relationship between the levels of *CHRM3*/M3R and MMP1 expression. To our knowledge, this represents the first report of differential expression of M3R in different stages of colon neoplasia.

## RESULTS

### Relative *CHRM3* mRNA expression in colon adenocarcinomas compared to matched adjacent normal colon

We initially sought to verify that *CHRM3*, the gene encoding M3R, was over-expressed in colon cancer and to explore whether this was associated with any important clinical characteristics. As shown in Table 1, compared to adjacent normal colon, *CHRM3* was over-expressed in 10 of 18 colon cancers (56%), a value consistent with those reported by others [2, 3]. In eight samples *CHRM3* expression was increased 2- to 128-fold compared to that in matched adjacent normal colon; the great variation in *CHRM3* expression most likely accounts for the failure to achieve statistical significance for the difference in *CHRM3* expression in colon cancer versus adjacent normal colon (*P* = 0.08).

**Table 1:** Levels of CHRM3 mRNA expression in adenocarcinoma relative to matched adjacent normal colon

Specimen Number	Anatomic Location	Tumor Stage	Tumor Differentiation	*CHRM3* mRNA (fold-change)
1	Left colon	T3N0M0	Moderate	0.32
2	Right colon	T4N0Mx	Well	0.35
3	Left colon	T3N0Mx	Moderate	0.57
4	Left colon	T2N0M0	Moderate	0.71
5	Left colon	T3N1Mx	Moderate	0.72
6	Right colon	T4aN0Mx	Moderate	0.73
7	Right colon	T3N2Mx	Well to Moderate	0.78
8	Left colon	T2N1Mx	Well	1.00
9	Right colon	T3N0M0	Poor	1.35
10	Left colon	T4bM1	Moderate	1.74
11	Right colon	T4N0Mx	Poor	2.80
12	Right colon	T3N1M1	Moderate	3.62
13	Right colon	T3N0Mx	Well	4.10
14	Right colon	T3N2bM1	Moderate	5.21
15	Left colon	T2N0Mx	Moderate to Poor	6.98
16	Right colon	T4aN2M1	Poor	24.57
17	Left colon	T3N1Mx	Moderate	31.05
18	Right colon	T3N0M1	Well	128.18

Levels of *CHRM3* mRNA expression were normalized to those of β_2_*-microglobulin*. Results represent the means of triplicate determinations and are expressed as fold-change relative to values obtained in adjacent normal colon tissue.

Several studies suggest important biological difference between cancers of the left and right colon [19–21]; we examined the relationship between *CHRM3* expression and the anatomic location, stage, and differentiation of colon cancer (Table 1). We observed no statistically significant relationship between *CHRM3* expression and anatomic location or tumor differentiation (*P* = 0.35 and 0.10, respectively). There was, however, a significant relationship between the level of *CHRM3* expression and the presence of colon cancer metastases; whereas metastases were absent in all 8 cancers lacking over-expression of *CHRM3*, metastatic disease was present in 5 of 10 individuals (50%) in whom *CHRM3* was over-expressed in the primary tumor (*P* = 0.04). This finding is consistent with our *in vitro* studies showing that treating human colon cancer cells with M3R agonists stimulates both cell migration and invasion, key features of tumor cells with metastatic capability [22–25].

### Relative M3R immunostaining in adenocarcinomas compared to adjacent normal colon

To analyze M3R protein expression, we interrogated 29 consecutive paraffin-embedded colon adenocarcinomas and adjacent normal colon epithelium using a specific anti-M3R antibody and immunoperoxidase staining. The specificity of the anti-M3R antibody was verified using colon tissue obtained from M3R-deficient mice with targeted deletion of *Chrm3* [15]. As shown in Supplementary Figure 1, a robust signal was observed in colon tissue from wild-type mice whereas no signal was observed in tissue from M3R-deficient animals or in control experiments performed without addition of the primary antibody.

**Figure 1:**
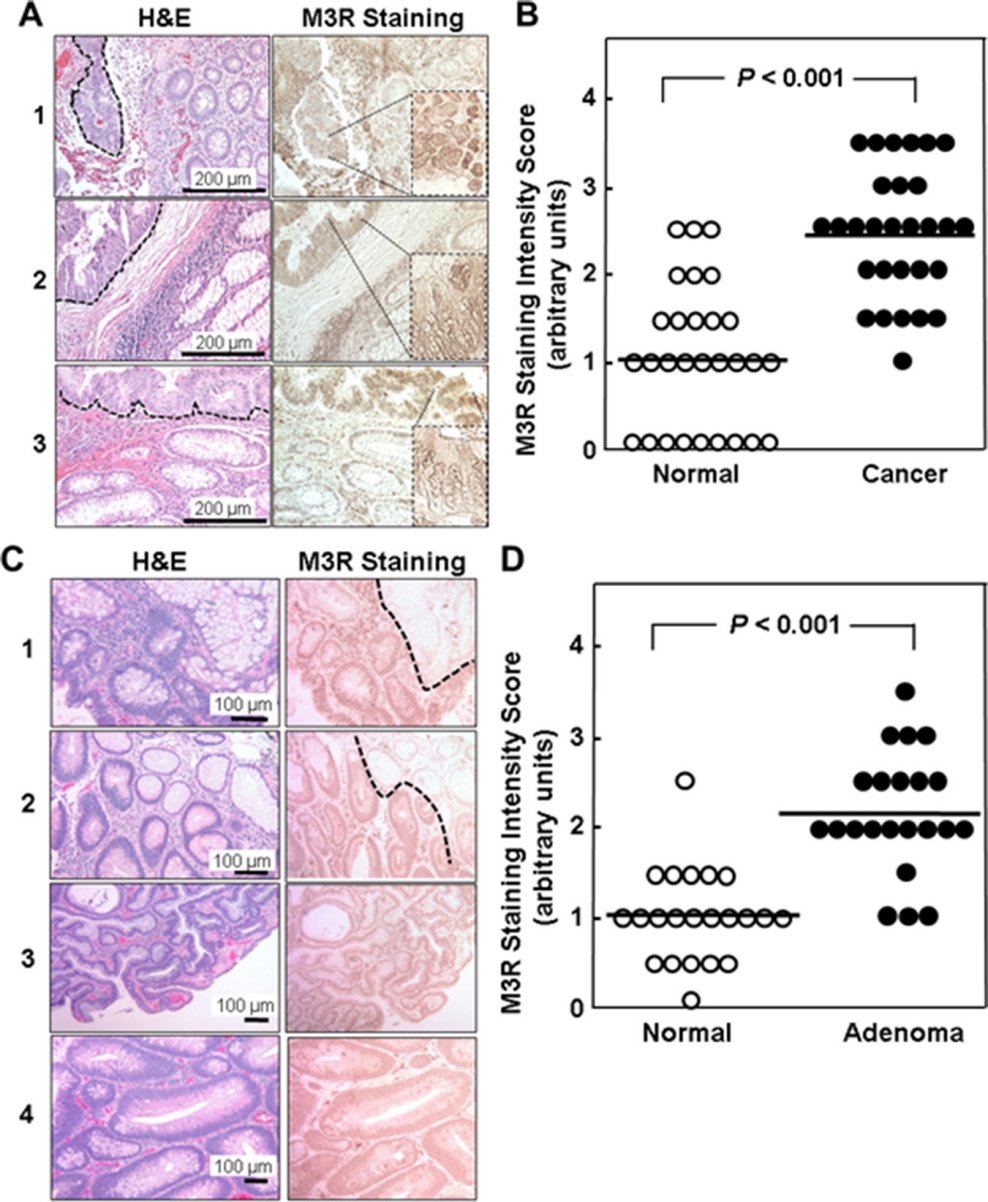
Relative M3R immunostaining in adenocarcinomas and adenomas compared to adjacent normal colon from the same patient (**A**) H&E and M3R immunoperoxidase staining is shown for three representative tissue samples. In normal colon, M3R is expressed weakly, primarily on basolateral surfaces of colonocytes. In well- and moderately-differentiated cancer (delineated by dashed lines in H&E sections) focal and diffuse M3R over-expression is seen in the cytoplasm and plasma membranes. (**B**). M3R staining intensity in 29 colon adenocarcinomas and adjacent normal colon was scored in 0.5 increments on a scale from 1 to 3 arbitrary units, where 0 represents absent and 3 maximal staining. Symbols represent individual tissue samples and horizontal bars represent means. (**C**) H&E and M3R immunoperoxidase staining is shown for four representative adenomas. In samples 1 and 2, normal colon epithelium present in the upper right hand corner of the images, delineated by dashed lines, shows less M3R staining compared to adenoma. (**D**) Staining intensity in 22 adenomas was scored on a scale from 1 to 3 arbitrary units in 0.5 increments where 0 is absent and 3 is maximal staining. Symbols represent individual tissue samples. Horizontal bars represent means.

It is noteworthy that the cellular distribution of M3R immunostaining was different in normal colon epithelium compared to that in malignant cells. Immunohistochemical analysis revealed weak M3R expression in normal colonocytes, primarily on basolateral surfaces (see examples in Figure 1A). In contrast, in colon cancer we observed both cytoplasmic and plasma membrane M3R staining (Figure 1A).

Compared to normal colon epithelium, mean M3R staining intensity was increased more than two-fold in colon cancer (1.03 ± 0.16 vs. 2.45 ± 0.14 arbitrary units, mean ± SE; *P* < 0.001); tumor M3R staining intensity exceeded that observed in paired adjacent normal epithelium in 25 of 29 samples (86%) (Figure 1B). In concert with the data shown in Table 1, these findings provide strong support for the concept that *CHRM3*/M3R expression is increased in colon cancer. As we observed for *CHRM3* mRNA expression, there was no statistically significant relationship between M3R immunostaining and anatomic location or tumor differentiation.

### Relative M3R immunostaining in adenomas compared to adjacent normal colon

Next, we sought to determine whether M3R over-expression is apparent at earlier stages of colon neoplasia. Based on the importance of M3R activation on colon cancer cell proliferation and survival, we hypothesized that increased M3R expression would occur early in the development of colon neoplasia. It is generally accepted that adenocarcinomas of the colon derive from pre-existing adenomas [24, 26]. Hence, we compared M3R expression in colon adenomas to that in matched samples of normal colon epithelium obtained from the same individual.

As shown in Figure 1C, we also observed increased M3R expression in adenomas compared to normal colon. In a set of 22 adenomas compared to normal colon, M3R immunostaining was significantly increased compared to adjacent normal colon (2.16 ± 0.14 vs. 1.02 ± 0.11 arbitrary units, mean ± SE; *P* < 0.001; Figure 1D). Although it was not possible to compare M3R expression directly in the progression of an adenoma to an adenocarcinoma, the two-fold increased M3R over-expression in adenomas (Figure 1D) was similar to that observed in adenocarcinomas (Figure 1B), consistent with the hypothesis that over-expression of M3R is an early step in colon neoplasia.

### Relative M3R immunostaining in lymph node and liver metastases compared to matched primary colon adenocarcinomas and normal colon epithelium

**Figure 2:**
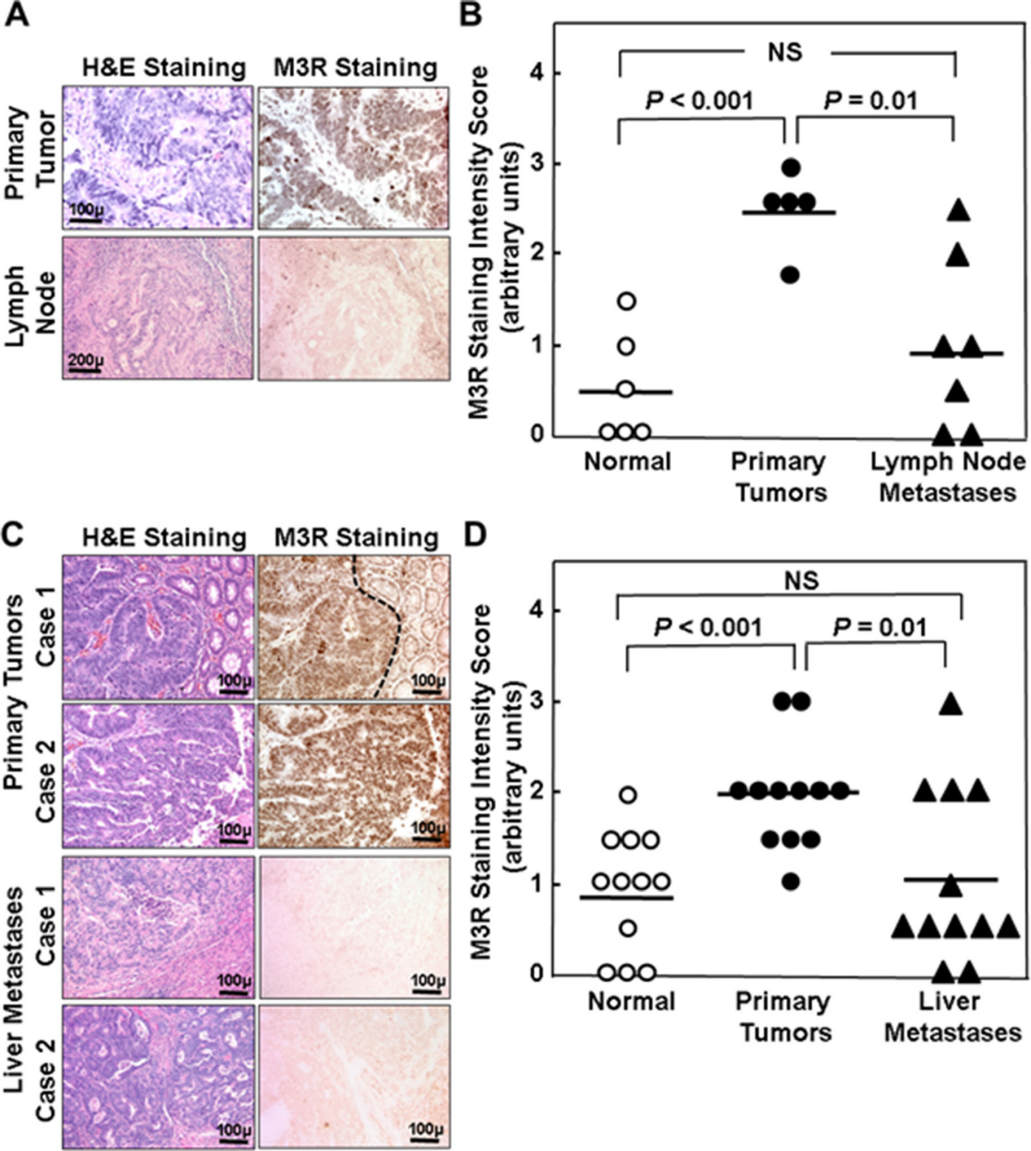
Relative M3R immunostaining in primary adenocarcinoma compared to lymph node and liver metastases from the same patient (**A**) H&E and M3R immunoperoxidase staining is shown for a representative set of primary adenocarcinoma and lymph node metastasis from the same patient. (**B**) Staining intensity for matched normal colon epithelium, primary adenocarcinoma, and lymph node metastases from seven different patients was scored on a scale from 1 to 3 arbitrary units in 0.5 increments where 0 is absent and 3 is maximal staining. Symbols represent individual tissue samples. Horizontal bars represent means. (**C**) H&E and M3R immunoperoxidase staining is shown for two representative sets of adenocarcinoma (top panels) and liver metastases (bottom panels) from the same two patients. Normal colon epithelium with weak M3R immunostaining can be seen to the right of the primary tumor in Case 1 (delineated by dashed line). (**D**) Staining intensity for matched normal colon epithelium, primary adenocarcinoma, and liver metastases from 12 different patients was scored on a scale from 1 to 3 arbitrary units in 0.5 increments where 0 is absent and 3 is maximal staining. Symbols represent individual tissue samples. Horizontal bars represent means.

We next compared M3R immunostaining in lymph node (Figure 2A, 2B) and liver metastases (Figure 2C, 2D) to that in matched normal colon and primary colon cancers (*n* = 7 for lymph node and *n* = 12 for liver metastases). As observed above (Figure 1B), the level of M3R immunostaining was increased approximately two-fold in primary tumors compared to normal colon epithelium, but M3R immunostaining was only minimally increased in lymph node and liver metastases (1.00 ± 0.36 vs. 0.50 ± 0.26 for lymph node metastases vs. normal colon and 1.04 ± 0.28 vs. 0.92 ± 0.19 for liver metastases vs. normal colon, mean ± SE; neither comparison was statistically significant) (Figures 2B and 2D). When we compared mean M3R immunostaining in all available samples, we again observed that immunostaining was the same as control (normal colon epithelium) in lymph node metastases and only minimally elevated in liver metastases (Table 2); as before, the only comparisons that achieved strong statistical significance were M3R staining in adenomas and adenocarcinomas versus normal colon epithelium.

**Table 2:** Results of M3R immunohistochemical staining analysis

Tissue Sample	Mean M3R Staining Score (au)	SE	Adjusted *P* versus Normal Colon Epithelium	Number of Samples
Normal colon epithelium	0.98	0.09	reference	64
Colon adenoma	2.16	0.14	< 0.001	22
Colon adenocarcinoma	2.51	0.11	< 0.001	42
Lymph node metastasis	0.88	0.34	1.0	8
Liver metastasis	1.32	0.28	0.19	17

^*^Adjusted *P* values were calculated based on a linear mixed model with Dunnett-Hsu's multiple comparison procedure.

### Relationship of *CHRM3*/M3R and *MMP1* mRNA over-expression in colon cancer

In human colon cancer cells, M3R activation induces a 30- to 50-fold increase in the levels of *MMP1* mRNA expression, an action that appears critical for colon cancer cell invasion [16, 23]. To explore the relationship between *CHRM3* and *MMP1* mRNA expression we used the same 18-sample dataset shown in Table 1. Compared to normal colon, we observed robust *MMP1* mRNA over-expression in 16 of the 18 cancer specimens (89%; Supplementary Table 2), but we were unable to identify a quantitative relationship between *CHRM3* and *MMP1* expression levels. Although the mean fold-change in *MMP1* mRNA level in cancers with increased *CHRM3* expression (samples 9–18) was almost 10-fold greater than those in cancers with reduced *CHRM3* expression (samples 1–7) (*MMP1* mRNA fold-change, 1042.5 ± 711.0 vs 119.5 ± 60.8; mean ± SE), due to considerable inter-sample variation and large standard errors this difference failed to achieve statistical significance.

Next, we used a specific anti-MMP1 antibody and immunoperoxidase staining to measure MMP1 protein expression in formalin-fixed paraffin-embedded tissue. Unlike membrane-anchored M3R, MMP1 is a secreted protein that can be washed away during tissue processing. Thus, as shown in Supplementary Figure 2, the detection of MMP1 staining was limited to peri-cellular surfaces and mucin vacuoles. Applying immunofluorescence to determine the relationship between M3R and MMP1 expression in normal colon, adenoma, and adenocarcinoma from the same individual appeared to show the redistribution of MMP1 staining from glandular lumens in normal colon to cell surfaces in cancer (Supplementary Figure 3). Although we could identify areas of intense over-expression of both M3R and MMP1 in adenoma and adenocarcinoma tissue (Supplementary Figure 3), we were unable to quantify the relationship between such changes consistently.

## DISCUSSION

This first report of differential expression of M3R in colon neoplasia provides useful information. First, it appears that increased M3R expression is an early feature of colon neoplasia – compared to adjacent normal colon, M3R expression is increased in adenomas to nearly the same extent as in adenocarcinomas. Second, increased expression of M3R in primary colon cancers appears to be associated with the presence of metastatic disease. This may reflect the *in vitro* observation that activation of M3R stimulates colon cancer cell invasion, a prelude to tumor dissemination [22, 23, 25]. Third, it appears that M3R expression is less important for the sustenance or progression of colon cancer metastases than for the growth and spread of primary colon neoplasia. We base this speculation on the observations that the levels of M3R expression in both lymph node and liver metastases are lower than in matched primary colon adenocarcinomas. Fourth, in addition to increased M3R expression in colon cancer we noted a change in the intracellular distribution of M3R immunostaining from being primarily associated with basolateral membranes to a broader distribution within cancer cells, including cytoplasmic localization. This finding is consistent with M3R transition from a quiescent plasma membrane-associated localization in normal colon epithelium to a more dynamic, functional role in cancer that likely involves M3R recycling. In addition to M3R expression serving as a surrogate marker for M3R activation [9] this suggests that the broader cellular distribution of immunostaining could provide an additional marker of M3R activation.

It appears that relying on *CHRM3* expression underestimates the degree to which M3R itself is over-expressed; whereas we found increased *CHRM3* mRNA expression in 56% of colon cancers compared to adjacent normal colon, we detected M3R protein over-expression in 86% of a larger set of tissue samples. This begs a question we cannot answer with certainty – Why does measuring *CHRM3* mRNA expression underestimate M3R over-expression in colon cancer? This, perhaps not surprisingly, weak correlation between gene and protein expression has been considered by others [27]. It is important to remember that protein abundance reflects a dynamic balance between mRNA processing and degradation as well as protein translation, localization, modification, and hydrolysis. Thus, *CHRM3* transcript abundance may not be the major determinant of M3R protein abundance. Factors like the actions of miRNA’s, the relative fragility of mRNA compared to protein, and others may also play a role [27, 28]. For example, substantial RNA degradation may occur between the time colon tissue is resected and frozen.

The experimental design used herein has several strengths. Chief amongst these is that whenever possible we compared normal colon, adenomas, adenocarcinomas, and metastases in matched tissue samples from the same individuals. Nonetheless, we recognize that this work has limitations. One consequence of studying tissue from the same individuals is that for lymph node and liver metastases we had relatively small sample sizes to work with. Another limitation is that ours was a retrospective analysis; thus, although increased *CHRM3* expression in the primary colon cancer appears to make it more likely metastases are present, we have not studied this prospectively.

MMP1, a matrix metalloproteinase associated with more aggressive tumors and whose expression is induced by M3R activation in human colon cancer cells, was almost uniformly over-expressed in adenocarcinomas compared to normal colon. Whereas the levels of *MMP1* over-expression were generally associated with those of M3R expression (*MMP1* and M3R were over-expressed in 89% and 86%, respectively, of colon cancers), both large inter-sample variations in *MMP1* expression and limited sample size contributed to our inability to identify a direct correlation between these events. It is also important to note that M3R activation, not *CHRM3*/M3R expression, drives the induction of MMP1 expression [23, 29]. So, even though *CHRM3*/M3R expression may be a surrogate for M3R activation and we detected a general association between *CHRM3* and *MMP1* expression, it is perhaps not surprising that this was insufficient to form the basis for a quantitative relationship between *CHRM3*/M3R and MMP1 expression.

In conclusion, we report the novel findings that M3 muscarinic receptors are expressed differentially in various stages of colon neoplasia, and that increased M3R expression in primary adenocarcinomas of the colon may be a biomarker for metastatic potential. We also identified changes in the cellular distribution of M3R in colon adenomas and carcinomas that are most likely indicative of activated M3R signaling, and receptor internalization and recycling. Overall, these observations are consistent with an emerging body of literature that highlights the importance of muscarinic receptor expression and activation of M3R signaling cascades in cancer progression [30].

## MATERIALS AND METHODS

### Human tissues

To examine M3R (*CHRM3*) and MMP1 gene and protein expression, we used archived pre-existing de-identified surgical specimens of colon cancer and adjacent normal colon epithelium (approved by the University of Maryland School of Medicine Institutional Review Board and the Baltimore VA Research and Development Committee). To ensure sufficient material for expression analysis and uniform distribution of immunohistochemical staining throughout a sample, we used only surgical tissue specimens (i.e. smaller, endoscopic biopsies were not used).

### Immunohistochemistry

Formalin-fixed paraffin-embedded tumor sections were immunostained using specific antibodies against both human and mouse M3R from Alomone Labs (Jerusalem, Israel) and against MMP1 from Santa Cruz (Dallas, TX), and following the vendors’ recommendations. Stained tumor sections were examined with a Nikon 80*i* photomicroscope at 200x magnification. Sections were reviewed and scored by a senior pathologist (CD) masked to tissue origin. To minimize variation, all tumor sections were examined and photographed using the same microscope settings.

### Quantitative RT-PCR (qPCR)

First-strand cDNAs were synthesized from 5 μg RNA (Superscript III First Strand Synthesis System for RT-PCR, Invitrogen). qPCR was performed using 50 ng cDNA, the SYBR Green PCR Master Mix (Applied Biosystems), and forward and reverse primers (final concentration 0.5 μM in sample volumes of 20 μl). Primers (Supplementary Table 1) were designed to span introns using the National Center for Biotechnology Information nucleotide database SIM-4 gene alignment program and on-line software (www.genscript.com/ssl-bin/app/primer). qPCR was performed using the 7900HT Fast System (ABI) with Power SYBR Green Master Mix (ABI). PCR conditions included 5 min at 95°C followed by 37 cycles of 95°C for 15 seconds, 60°C for 20 seconds, and 72°C for 40 seconds and a final cycle at 95°C for 15 seconds, 60°C for 15 seconds, and 95°C for 15 seconds. PCR data were analyzed using ABI instrument software SDS 2.1. Expression of *CHRM3* and *MMP1* was normalized to β2*-microglobulin* (*B2M*), a preferable housekeeping gene for analysis of colon cancer.[31] Quantitative qPCR data were evaluated using the comparative *C*_T_ (2^–ΔΔ*C*T^) method.[32]

### Statistical analysis

To compare fold-changes in *CHRM3* expression we used the Wilcoxon signed rank test on log-transformed data. Fisher's exact test was used to analyze contingency tables for small sample sizes. To calculate adjusted *P* values for comparisons of M3R immunostaining we employed linear mixed model analysis on the ranks of staining scores and the Dunnett-Hsu method for multiple comparisons. Statistical significance was set at *P* < 0.05.

## SUPPLEMENTARY MATERIALS FIGURES AND TABLES


