# Supplementary material for: Differential expression of M3 muscarinic receptors in progressive colon neoplasia and metastasis
Source: Oncotarget. 2017 Feb 18;8(13):21106–14. doi: 10.18632/oncotarget.15500 (PMC5400569; doi:10.18632/oncotarget.15500)
Supplement: Supplementary file 1 [file oncotarget-08-21106-s001.pdf]

Differential expression of M3 muscarinic receptors in progressive colon neoplasia and metastasis

Supplementary Materials

Supplemental Table 1: qPCR primer sequences

Protein	Gene	Forward	Reverse
Matrix metalloproteinase-1	*MMP1*	AACTGCCAAATGGGCTTG	CCGTGTAGCACATTCTGTCC
M3R	*CHRM3*	TGGTTTGATGCTCCTACCTG	AGGACAGAGGAGTGGACCAG
Beta-2-microglobulin	*B2M*	GAGGCTATCCAGCGTACTCC	ATGGATGAAACCCAGACACA

Supplemental Table 2: *MMP1* expression in adenocarcinoma relative to matched adjacent normal colon

Specimen Number	Anatomic Location	Tumor Stage	Tumor Differentiation	*MMP1* mRNA (fold-change)
1	Left colon	T3N0M0	Moderate	5.0
2	Right colon	T4N0Mx	Well	196.6
3	Left colon	T3N0Mx	Moderate	20.3
4	Left colon	T2N0M0	Moderate	0.1
5	Left colon	T3N1Mx	Moderate	5.8
6	Right colon	T4aN0Mx	Moderate	179.1
7	Right colon	T3N2Mx	Well to Moderate	429.1
8	Left colon	T2N1Mx	Well	86.6
9	Right colon	T3N0M0	Poor	3.6
10	Left colon	T4bM1	Moderate	6.6
11	Right colon	T4N0Mx	Poor	31.4
12	Right colon	T3N1M1	Moderate	11.6
13	Right colon	T3N0Mx	Well	82.2
14	Right colon	T3N2bM1	Moderate	0.7
15	Left colon	T2N0Mx	Moderate to Poor	3465.6
16	Right colon	T4aN2M1	Poor	1.7
17	Left colon	T3N1Mx	Moderate	6657.5
18	Right colon	T3N0M1	Well	163.8

MMP1 expression was normalized to that of β2-*microglobulin*. Results represent the means of triplicate determinations and are expressed as fold-change relative to values obtained in adjacent normal colon tissue. Specimen numbers correspond to those for *CHRM3* expression shown in Table 1.

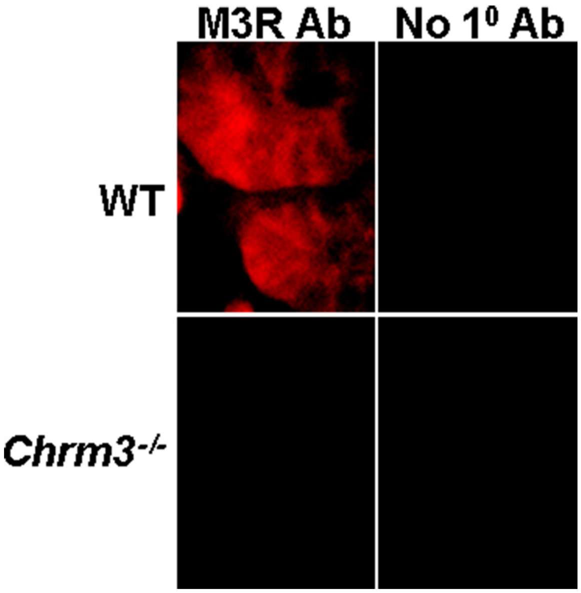

Supplementary Figure 1: Anti-M3R antibody specificity. The antibody (Ab) was tested using colon sections from WT and M3R-deficient *Chrm3*$^{-/-}$ mice. Robust immunostaining was observed only when primary (1°) anti-M3R antibody (1:100) and secondary antibody (Alexa-647, Invitrogen) were used in tissue from WT mice.

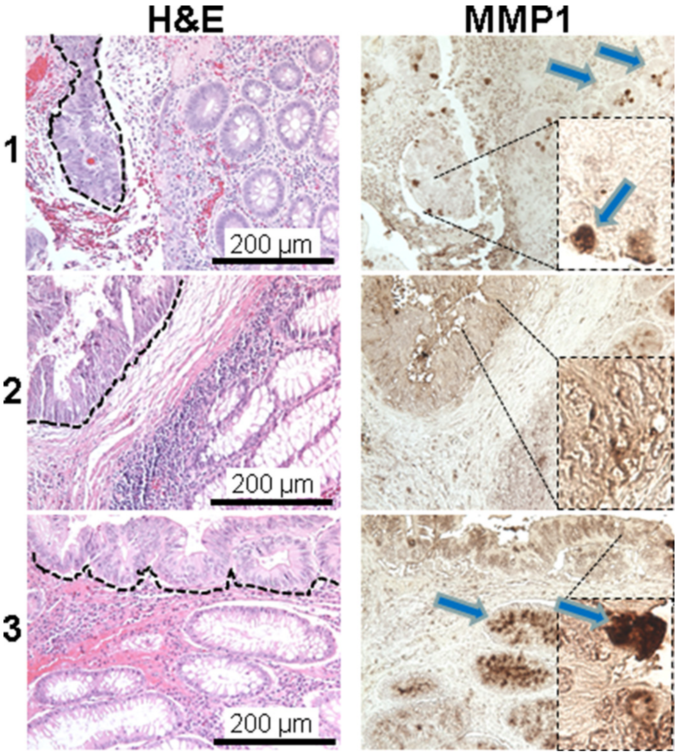

Supplementary Figure 2: MMP1 expression in colon cancer and adjacent normal epithelium. H&E and MMP1 immunoperoxidase staining of representative tissue specimens. In normal colon, MMP1 staining is seen on apical surfaces, glandular lumens and mucin vacuoles (arrows). With a paucity of gland formation in cancer, MMP1 over-expression is limited to peri-cellular surfaces and mucin vacuoles (insets).

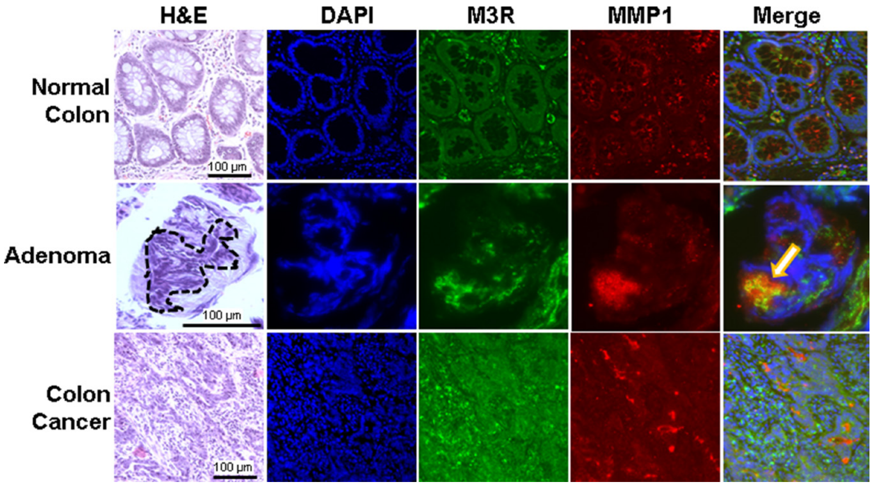

Supplementary Figure 3: Increased M3R and MMP1 immunostaining in adenoma and colon cancer compared to normal colon from the same patient. Representative images of normal colon, adenoma (neoplasia delineated by dashed line in H&E) and colon cancer. DAPI nuclear stain (blue), M3R (green-Alexa Fluor 488), MMP1 (red-Alexa Fluor 594), and merged immunostaining are shown. Arrow indicates area of intense over-expression of both M3R and MMP1 in adenoma. Relative M3R and MMP1 staining intensity: adenoma > colon cancer >> normal colon.
